# Synthesis of sialoglycopolypeptide for potentially blocking influenza virus infection using a rat α2,6-sialyltransferase expressed in BmNPV bacmid-injected silkworm larvae

**DOI:** 10.1186/1472-6750-9-54

**Published:** 2009-06-05

**Authors:** Makoto Ogata, Makoto Nakajima, Tatsuya Kato, Takakiyo Obara, Hirokazu Yagi, Koichi Kato, Taichi Usui, Enoch Y Park

**Affiliations:** 1 Department of Applied Biological Chemistry, Faculty of Agriculture, Shizuoka University, Ohya 836, Suruga-ku, Shizuoka 422-8529, Japan; 2 Graduate School of Pharmaceutical Sciences, Nagoya City University, 3-1 Tanabe-dori, Mizuho-ku, Nagoya 467-8603, Japan; 3 Institute for Molecular Science and Okazaki Institute for Integrative Bioscience, National Institutes of National Science, 5-1 Higashiyama Myodaiji, Okazaki 444-8787, Japan; 4 GLYENCE Co., Ltd., 2-22-8 Chikusa, Chikusa-ku, Nagoya 464-0858, Japan; 5 The Glycoscience Institute, Ochanomizu University, 2-1-1 Ohtsuka, Bunkyo-ku, Tokyo 112-8610, Japan; 6 Integrated Bioscience Section, Graduate School of Science and Technology, Shizuoka University, Ohya 836, Suruga-ku, Shizuoka 422-8529, Japan

## Abstract

**Background:**

Sialic acid is a deoxy uronic acid with a skeleton of nine carbons which is mostly found on cell surface in animals. This sialic acid on cell surface performs various biological functions by acting as a receptor for microorganisms, viruses, toxins, and hormones; by masking receptors; and by regulating the immune system. In order to synthesize an artificial sialoglycoprotein, we developed a large-scale production of rat α2,6-sialyltransferase (ST6Gal1). The ST6Gal1 was expressed in fifth instar silkworm larval hemolymph using recombinant both cysteine protease- and chitinase-deficient *Bombyx mori *nucleopolyhedrovirus (BmNPV-*CP*^-^-*Chi*^-^) bacmid. The expressed ST6Gal1 was purified, characterized and used for sialylation of asialoglycopolypeptide. We tested the inhibitory effect of the synthesized α2,6-sialoglycopolypeptide on hemagglutination by *Sambucus nigra *(SNA) lectin.

**Results:**

FLAG-tagged recombinant ST6Gal1 was expressed efficiently and purified by precipitation with ammonium sulphate followed by affinity chromatography on an anti-FLAG M2 column, generating 2.2 mg purified fusion protein from only 11 silkworm larvae, with a recovery yield of 64%. The purified ST6Gal1 was characterized and its *N*-glycan patterns were found to be approximately paucimannosidic type by HPLC mapping method. Fluorescently-labelled *N*-acetyllactosamine (LacNAc) glycoside containing dansyl group was synthesized chemo-enzymatically as high-sensitivity acceptor substrate for ST6Gal1. The acceptor substrate specificity of the enzyme was similar to that of rat liver ST6Gal1. The fluorescent glycoside is useful as a substrate for a highly sensitive picomole assay of ST6Gal1. Asialoglycopolypeptide was regioselectively and quantitatively sialylated by catalytic reaction at the terminal Gal residue to obtain α2,6-sialoglycopolypeptide using ST6Gal1. The α2,6-sialoglycopolypeptide selectively inhibited hemagglutination induced by *Sambucus nigra *(SNA) lectin, showing about 780-fold higher affinity than the control fetuin. Asialoglycopolypeptide and γ-polyglutamic acid did not affect SNA lectin-mediated hemagglutination.

**Conclusion:**

The recombinant ST6Gal1 from a silkworm expression system is useful for the sialylation of asialoglycopeptide. The sialylated glycoprotein is a valuable tool for investigating the molecular mechanisms of biological and physiological events, such as cell-cell recognition and viral entry during infection.

## Background

Sialic acids are distributed in a variety of glycolipids and glycoproteins, often existing at the non-reducing termini of carbohydrate chains. Sialic acids play important roles in various biological and physiological events [1,2]. Sialic acid is added to the terminal sugar of glycoproteins and glycolipids by sialyltransferase (SiaT) enzymes. The sialic acid that is added to a galactose (Gal) can be bound either to the hydroxyl attached to carbon-3 of Gal to form an α2,3 glycosidic linkage, or to the hydroxyl group attached to carbon-6 to form an α2,6 glycosidic linkage. Weinstein et al., [3] reported that the ST6Gal1 (EC 2. 4. 99. 1) generates a α2-6 linkage of sialic acid on the non-reducing, terminal Galβ1-4GlcNAc residues of oligosaccharides and glycoconjugates. Carbohydrate structures containing Neu5Acα2,6 residues play critical roles in cell-cell recognition and influenza virus infection [2].

Several systems for expression of recombinant proteins are available, based on bacteria [4,5], yeast [6], insect [7-9] and mammalian cells [10]. Since bacterial system recombinant proteins are often insoluble and inactive, alternative expression systems to obtain soluble and active recombinant proteins are required. Insect cells infected with recombinant baculovirus have been used for high-level expression of recombinant proteins [9,11], partly because these insect cells are capable of posttranslational modifications similar to mammalian cells, and because of high-level protein expression. Recombinant baculoviruses are used to infect insect cells, such as Sf9 cells, and recombinant proteins are recovered from infected cells. However, reactor performance, reactor design, and medium development still require improvements to increase the yield of recombinant proteins. For example, serial passaging and preparation of a large amount of recombinant baculovirus for infection of insect cells are drawbacks of the baculovirus-insect cell expression system.

An alternative to the baculovirus expression system is silkworm larvae expression. Recently, a *B. mori *nucleopolyhedrovirus (BmNPV) bacmid system was developed [12] as a shuttle vector that can be replicated in *Escherichia coli*, *B. mori *cells and silkworm larvae. This enables more rapid gene expression in silkworm than in conventional baculovirus expression systems. Moreover, a cysteine protease-deficient BmNPV (BmNPV-*CP*^-^) bacmid [13] and both cysteine protease- and chitinase-deficient BmNPV (BmNPV-*CP*^-^-*Chi*^-^) bacmids [14] have been developed for the efficient production of gene products from silkworms. Using this bacmid, human IgG [15], (pro)renin receptor [16], and glycosyltransferase [17] were expressed successfully. The protein expression of these bacmids is higher than that of wild-type BmNPV bacmid because of significant decreases in silkworm liquefaction and proteolytic degradation of expressed proteins.

In this study, we have successfully expressed a functional rat ST6Gal1 in silkworm larvae, established efficient procedures for large-scale purification of the recombinant enzyme. Furthermore, a novel synthetic substrate, fluorescently-labelled disaccharide glycoside was chemo-enzymatically synthesized for the ST6Gal1 assay. Characterization and identification of *N*-glycans of the purified recombinant rat ST6Gal1 from the silkworm larval hemolymph are also reported. The recombinant ST6Gal1 was used as a key enzyme for the synthesis of an artificial sialoglycopolymer for potentially blocking influenza virus infection.

## Results

### Expression of recombinant ST6Gal1 in bacmid-injected silkworm larval hemolymph

**Figure 1:**
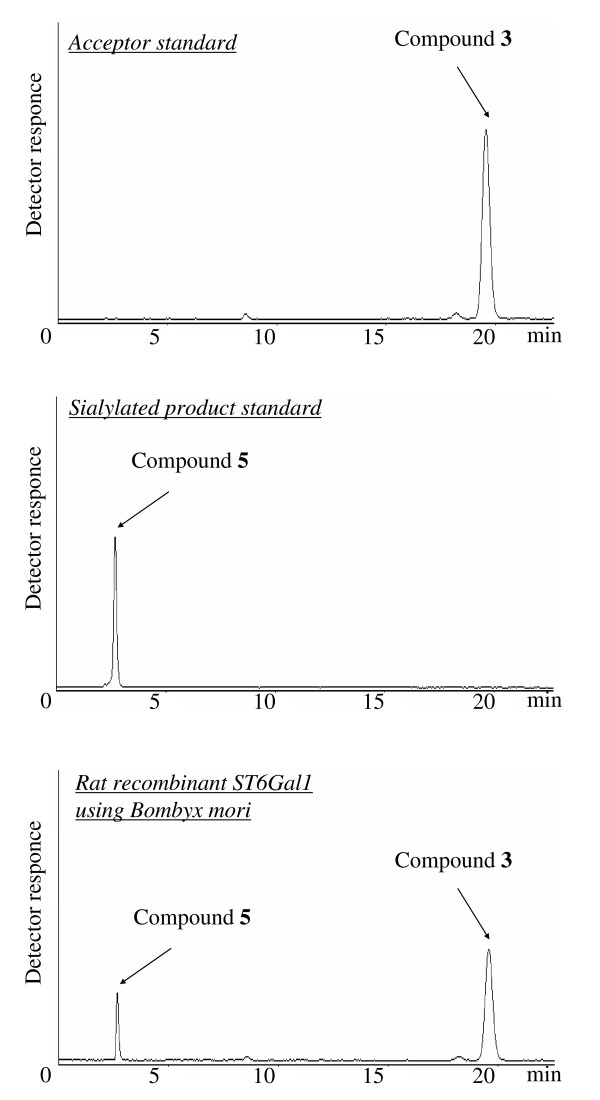
**Sialylation of compound 3 as acceptor substrate by recombinant ST6Gal1**. Detection of sialylated product **5 **was performed by fluorescent-HPLC as described in *Materials and methods*.

BmNPV-*CP*^-^-*Chi*^-^/ST6Gal1 with bombyxin (bx) signal peptides was constructed for secretion of ST6Gal1 into the silkworm larval hemolymph. Hemolymph was sampled at 1.5–7.5 d.p.i. and subjected to sialyltransferase activity assay. Compound **3 **was used as a novel synthetic substrate for the fluorescence assay of sialyltransferase [see Additional file 1]. We first investigated the action of recombinant ST6Gal1 on **3 **and CMP-Neu5Ac. When recombinant ST6Gal1 was incubated with **3** and CMP-Neu5Ac, it exclusively produced 5-(5-dimetylaminonaphthalene-1-sulfonyl-2-(2-aminoethoxy))ethyl β-Neu5Acα2,6LacNAc (compound **5**) as described in Additional file 1. The resulting compound **5 **was used as an authentic sample for enzyme assays. The ST6Gal1 assay used HPLC to determine the fluorescence of **5 **produced from **3 **and CMP-Neu5Ac by recombinant ST6Gal1 (Fig. 1).

**Figure 2:**
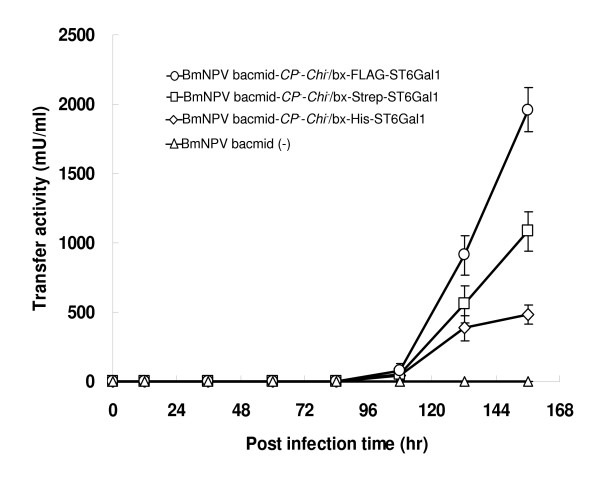
**Expression levels of recombinant ST6Gal1s in silkworm larval hemolymph**. Three types of BmNPV bacmids were injected directly into 1^st ^day fifth-instar silkworm larvae. The expression of the fusion protein in silkworm larvae was confirmed using a sialyltransferase activity assay.

Rat ST6Gal1 was cloned as *N*-terminal His-, Strep- and FLAG-tagged fusion proteins. When the three fusion proteins were tested in the sialyltransferase activity assay, FLAG-tagged ST6Gal1 (1960 mU/ml at 6.5 d.p.i.) had the highest expression level, which was 1.8- or 4.1-hold higher than that of Strep- or His-tagged ST6Gal1 (1083 mU/ml or 480 mU/ml at 6.5 d.p.i.), respectively (Fig. 2). No activity was observed in mock-injected silkworm larval hemolymph (Fig. 2).

### Purification of recombinant FLAG-tagged ST6Gal1 expressed in silkworm larval hemolymph

**Figure 3:**
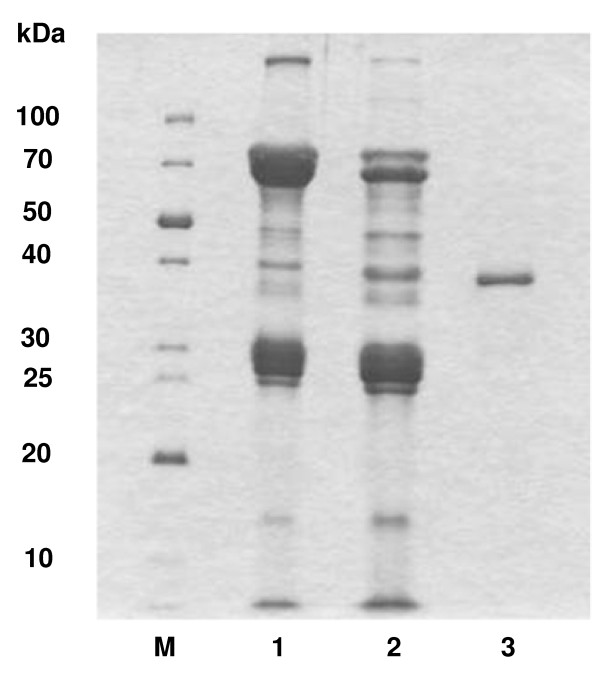
**SDS-PAGE of recombinant FLAG-tagged ST6Gal1 expressed in silkworm larval hemolymph**. Lane M, marker proteins, Lane 1, silkworm larval hemolymph (crude enzyme), Lane 2, ammonium sulphate precipitates, Lane 3, elution from anti-FLAG M2 affinity (purified recombinant enzyme).

**Figure 4:**
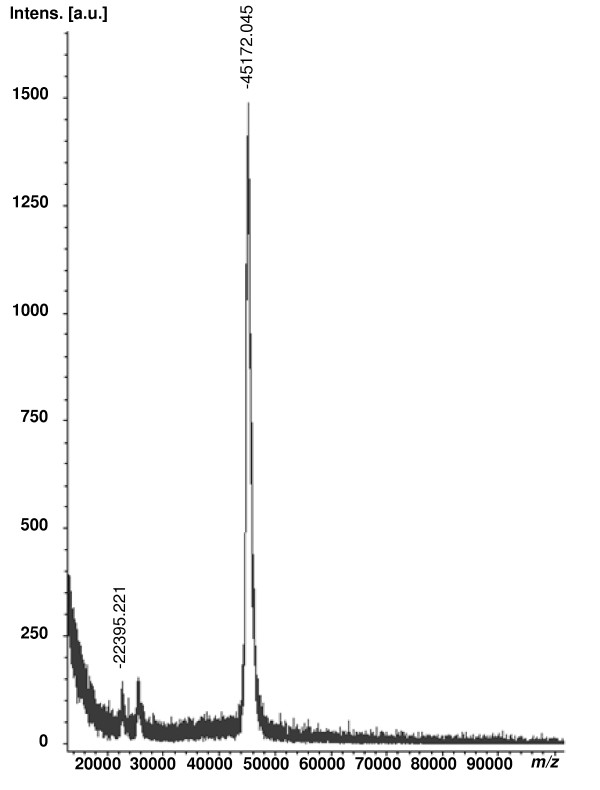
**MALDI-TOF mass spectrometry of recombinant FLAG-tagged ST6Gal1**. Each sample was dissolved in 0.1% TFA: acetonitrile (2:1 v/v) and mixed with the matrix solution (1:4 v/v). The mixture (1 μl) was put on a stainless target and crystallized at room temperature. A mass calibration procedure was employed prior to the analysis of a sample using protein calibration standards I (Bruker Daltonics, Germany). The MALDI-TOF mass spectrum was acquired on an AutoFlex (Bruker Daltonics, Germany) and measured in linear mode using 20-kV ion acceleration without postacceleration. The spectrum was recorded at a detector voltage of 1.65 kV and was the averaged results of at least 300 laser shots. SDHB was used as the matrix.

The purification of FLAG-tagged ST6Gal1 from 4.5 ml of silkworm larval hemolymph is summarized in Table 1. Twofold diluted hemolymph containing 9.0 units of FLAG-tagged ST6Gal1 (0.016 U/mg protein) was saturated with 25–70 ammonium sulphate and the resulting precipitate protein was dissolved in 50 mM MOPS buffer (pH 7.5) containing 150 mM NaCl and 0.02 Triton X-100. In this fraction 7.2 units of FLAG-tagged ST6Gal1 (0.069 U/mg protein) were obtained with a yield of 80. After removal of the ammonium sulphate with a Sephadex G-25M PD-10 column, the enzyme was applied to an anti-FLAG M2 column. Most of the sialyltransferase activity adsorbed to the affinity column and was eluted by elution with same buffer containing 100 μg/ml FLAG peptide. In this final purification step, 5.8 units of FLAG-tagged ST6Gal1 (2.6 U/mg protein) was obtained in a high yield of 64 (over 150-fold) (Table 1). FLAG-tagged ST6Gal1 gave a single band on SDS-PAGE and isoelectric focusing with an apparent mass of 40 kDa (Fig. 3) and a pI of 6.3 (data not shown). The MALDI-TOF mass spectrum gave a main peak at *m/z *45172 (Fig. 4).

**Table 1:** Purification of a recombinant FLAG-tagged ST6Gal1 from silkworm larval hemolymph

	Total activity (U^a^)	Total protein (mg)	Specific activity (U/mg protein)	Recovery yield (%)	Purification (fold)
Crude enzyme (larval hemolymph)	9.0	550.0	0.016	100	1
(NH_4_)_2_SO_4 _(25–70%)	7.2	105.0	0.07	80	4
Anti-FLAG M2 affinity	5.8	2.2	2.60	64	159

^a ^One unit of activity is defined as 1 μmol/min of transfer product **5 **formed from compound **3 **as acceptor substrate under the condition of 37°C and pH 7.4. Product **5 **and compound **3 **were measured by HPLC-based assay described in Materials and Methods.

### Characterization of the purified recombinant FLAG-tagged ST6Gal1

The optimum temperature of the purified enzyme was determined by performing the standard assay in the range 4 to 80°C. Maximum activity was observed at 37°C for the purified enzyme. The enzyme was stable below 20°C but was rapidly inactivated at temperatures above 20°C. The effect of pH on the activity of the purified enzyme was studied in various buffers in the range of pH 2 to 10. The optimum was pH 6.0. The purified enzyme was relatively stable over a wide range of pH, and was especially stable in the range of pH 5 to 9.

The kinetic parameters for the transfer reaction of fluorescently-labelled disaccharide glycosides by the purified enzyme were determined by HPLC assays. The enzyme acted preferentially on LacNAc glycoside over Lac glycoside. Thus, the *k*_cat_/*K*_m _value of LacNAc glycoside was 39-fold higher than that of Lac glycoside (Table 2).

**Table 2:** Kinetic parameter for transfer reaction of acceptor substrates

Sugar moiety	K_*m *_(mM)	*V*_*max *_(mM/min)	*k*_*cat *_(s^-1^)^a^	*k*_cat_/K_*m *_(mM^-1^s^-1^)
LacNAc β-R^b^	0.92	0.17	1.13	1.23
Lactose β-R	2.80	0.013	0.087	0.031

^a ^*k*_*cat *_= *V*_max_/E, where E denote amount of enzyme.

^b ^R = 5-(5-Dimetylaminonaphthalene-1-sulfonyl-2-(2-aminoethoxy))ethyl

**Figure 5:**
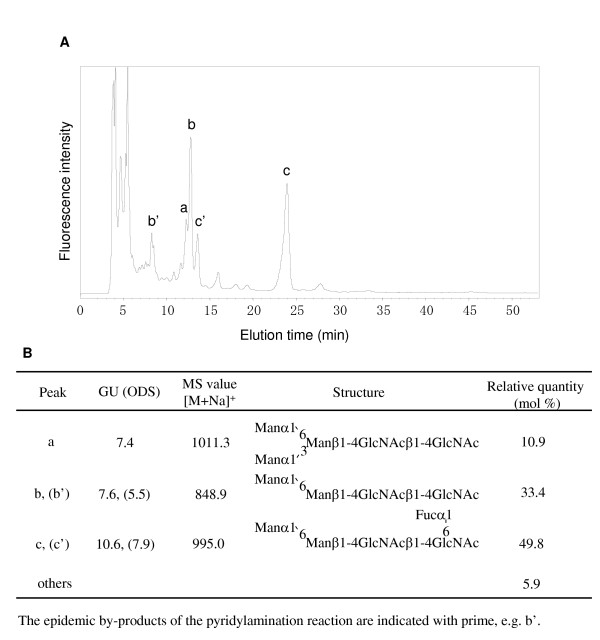
***N*-Glycosylation profile of recombinant FLAG-tagged ST6Gal1 and proposed structure of PA-oligosaccharides**. **A**, *N*-Glycosylation profile of recombinant FLAG-tagged ST6Gal1 on an ODS column. The epidemic by-products of pyridylamination reaction are indicated with prime, e.g. b'. **B**, The proposed structure of PA-oligosaccharides obtained using recombinant FLAG-tagged ST6Gal1 expressed in silkworm larval hemolymph.

The detailed chromatogram and structure of the oligosaccharides are shown in Fig. 5. Recombinant FLAG-tagged ST6Gal1 produced non-reducing terminal α-mannosyl groups with fucose (49.8) connected to GlcNAc near to Asn. The yield of non-reducing terminal α-mannosyl sugars was 94.1, including antennary sugars 10.9. An unidentified oligosaccharide might be included among the others (5.9) in Fig. 5B.

### Synthesis of α2,6-sialoglycopolypeptide as glycoprotein mimetics using recombinant ST6Gal1

**Figure 6:**
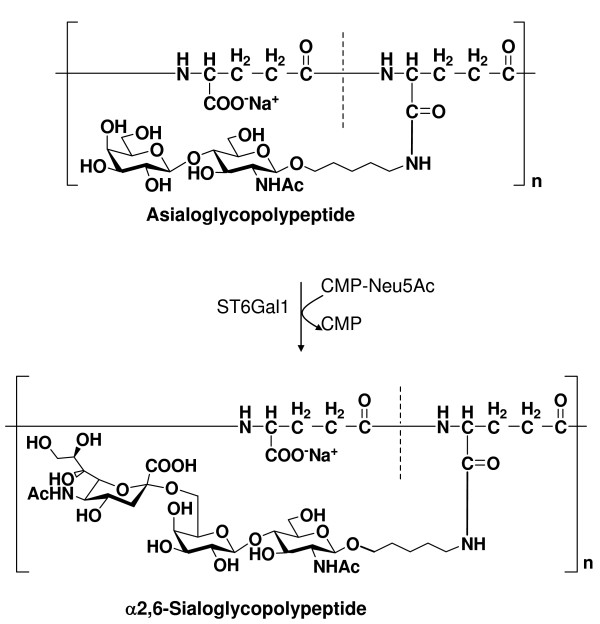
**Enzymatic synthesis of the artificial Sialoglycopolypeptide**. α2,6-Sialoglycopolypeptide was enzymatically synthesized from asialoglycopolypeptide using recombinant ST6Gal1. A mixture containing 5.0 mg of asialoglycopolypeptide, 16.0 mM CMP-β-Neu5Ac, 80 mU/ml of the purified FLAG-tagged ST6Gal1, 2.5 mM MnCl_2_, 0.1 BSA and 10 U/ml of calf intestine alkaline phosphatase (Boehringer-Mannheim, Mannheim, Germany) in 50 mM MOPS buffer (pH 7.4) was incubated at 37°C for 48 h in a total volume of 1.0 ml. After heating at 100°C followed by centrifugation, the supernatant from the reaction mixture was directly loaded onto a Sephadex G-25M PD-10 column equilibrated with 100 mM PBS (pH 7.4).

Asialoglycopolypeptide with a γ-PGA backbone was sialylated to a sialoglycopolypeptide carrying Neu5Acα2,6LacNAc, using recombinant ST6Gal1 from silkworm hemolymph as previously described [18-20] (Fig. 6). After separation with a Sephadex G-25M PD-10 column, the target glycopolypeptide was obtained. The structures of the synthesized α2,6-sialoglycopolypeptide were confirmed by ^1^H-, ^13^C-NMR and chemical analyses, according [19]. The ^1^H-NMR spectrum also showed that the degree of sialylation was quantitative from the integration data of the proton signals (Table 3) [21]. The degree of substitution of neutral sugar derivatives (NS) and sialyl sugar derivatives (Sia), based on the DP of γ-PGA was 100 (Table 3). These results indicated that the FLAG-tagged ST6Gal1 regioselectively transfers Neu5Ac to the non-reducing terminal of type II sugar chains, such as Galβ1-4GlcNAc residues.

**Table 3:** Inhibition of SNA lectin hemagglutination by the artificial sialoglycopolypeptide as glycoprotein mimetics

Entry compounds	kDa	NS^d ^(%)	Sia^h ^(%)	IC_50_^i ^(nM)
γ-Polyglutamic acid (γ-PGA)	990^a^	-	-	N.E.^*j*^
Asialoglycopolypeptide	2100^b^	40^e ^(33^f^)	-	N.E.
α2,6-Sialoglycopolypeptide	2800^b^	0	40 (39)	0.94
Fetuin (Sialoglycoprotein)	48^c^	N.D.^g^	(13.5)	730

^a ^Provided by Meiji Seika Kaisha, Ltd. (Tokyo, Japan).

^b ^Estimated from degree of substitution of asialo- or sialo-sugar derivatives based on DP of γ-PGA as 100%, which was calculated from ^1^H NMR data at 25°C.

^c ^Neutral sugar derivatives substituted

^d ^Degree of substitution of asialo- or sialo-sugar derivatives based on DP of γ-PGA as 100%, which was calculated from ^1^H NMR data at 25°C.

^e ^Degree of substitution of asialo- or sialo-sugar derivatives based on DP of γ-PGA as 100%, which was calculated from two types of HPLC analys (ABEE and DMB methods). All data normalized to those of ^1^H NMR.

^f ^Not determined

^g ^Sialyl sugar derivatives substituted (= Sia contents)

^h ^Minimum concentrations required for complete inhibition of hemagglutination

^i ^No effect means that inhibitory effect was not observed up to 200 nM of γ-PGA or asialoglycopolypeptide

### Hemagglutination inhibition assay with artificial sialoglycopolypeptides

Various substances carrying α2,6-sialooligosaccharide glycosides are known to inhibit hemagglutination by binding to SNA lectin [22]. We tested the inhibitory effect on hemagglutination by SNA lectin of the synthesized α2,6-sialoglycopolypeptide, using recombinant enzyme (Table 3). The α2,6-sialoglycopolypeptide prominently inhibited hemagglutination by SNA lectin. The affinity of α2,6-sialoglycopolypeptide for the SNA lectin was 780-fold higher than that of the control fetuin. Both the asialoglycopolypeptide and γ-polyglutamic acid itself did not caused any SNA lectin-mediated hemagglutination (Table 3).

## Discussion

Functional rat recombinant ST6Gal1 was expressed using a bacmid system. The amount of recombinant protein secreted from silkworm larvae was 2.0 U/ml (Fig. 2), which was higher than that obtained from other expression systems. For example, the expression level of secreted rat recombinant ST6Gal1 using Sf9 insect cells was reported as 0.03 U/ml [23], mammalian ST6Gal1 using yeast *Pichia pastoris *was reported as 0.3 × 10^-3 ^U/ml [24] and bacterial ST6Gal1 using *Photobacterium damsela *JT0160 cell was reported at 0.55 U/ml [25]. In addition, we produced various *N*-terminal-tagged ST6Gal1 using our silkworm expression system. FLAG-tagged ST6Gal1 had a high expression level, which was 4.1-fold higher than that of His-tagged ST6Gal1 (Fig. 2). Some investigators have reported the increase of expression levels of recombinant proteins when *N*-terminal sequences were changed [26-28]. Most likely, the polar character of the *N*-terminal sequence is contributing to the expression level of recombinant protein secreted from silkworm larvae.

The expressed FLAG-tagged ST6Gal1 was effectively purified by precipitation with ammonium sulphate followed by affinity chromatography. The purified protein reached 200 μg/larva (Table 1). This highly purified enzyme might be used for studies such as NMR or X-ray diffraction. Recombinant protein produced by BmNPV-*CP*^-^-*Chi*^-^/FLAG-tagged ST6Gal1 with bx signal peptides infected silkworm, was comparable to native rat liver ST6Gal1 with regard to substrate specificity and stability. FLAG-tagged ST6Gal1 lacks an *N*-terminal hydrophobic region found in the wild type protein, as previously reported by [29]. The *K*_m _of the purified recombinant enzyme (0.92 mM) against Galβ1,4GlcNAcβ-R (compound **3**, see Additional file 1) was similar to that those of rat liver ST6Gal1 [30] (Table 2). Furthermore, recombinant protein expressed in silkworm is *N*-glycosylated. Most (94%) of the *N*-glycan patterns of secreted recombinant proteins produced in the silkworm larvae were nearly paucimannosidic type structures, such as Man_3_GlcNAc_2 _or Man_2_GlcNAc_2 _(± Fuc) (Fig. 5B). *N*-glycosylation is known to be responsible for solubility and stability of glycoproteins. Recombinant protein expressed using silkworm larvae has a high solubility in buffer. Moreover, no significant loss of enzyme activity was observed upon storage for 3 month at 4°C.

In addition, we also established a convenient synthesis for fluorescently-labelled LacNAc glycoside as a high-sensitivity acceptor substrate for the assay of ST6Gal1 (see Additional file 1). LacNAc and lactose residues were enzymatically connected to 2-(2-trifluoroasetamidoethoxy)ethanol to produce the disaccharide glycosides. Recently, we found that endo-β-glycosidase, a kind of cellulase from *T. reesei*, catalyzes two reaction types, transglycosylation and condensation [31]. This enzymatic catalysis was harnessed for the synthesis of spacer-linked LacNAc and lactose glycosides. The efficiency of the reaction is not always high, but this method has two advantages: first, the excess of unreacted LacNAc substrate, which is a valuable substrate, is recovered by straightforward column chromatography and can be reutilized for synthesis; and second, the *O*-glycosylation process stereospecifically gives only the β-glycoside without the need for protection or deprotection steps [32]. After deacylation of the resulting disaccharide glycosides, the amino function was easily fluorescently-labelled. Especially, the use of fluorescently-labelled LacNAc glycoside enabled routine picomole assay of ST6Gal1. Furthermore, fluorescently-labelled glycosides with spacer structure containing ether group, such as 2-(2-aminoethoxy)ethanol have an extremely high solubility in water, as compared with alkyl spacer, such as 5-amino-1-pentanol [32].

We have already reported that sialoglycopolypeptides with γ-polyglutamic acid backbones can be used as a scaffold in the synthesis of multivalent inhibitors of the influenza viruses [19]. Thus, the recombinant enzyme was applied to the conversion of asialoglycopolypeptide to α2,6-sialoglycopolypeptide. The asialoglycopolypeptide was quantitatively sialylated to obtain sialoglycopolypeptide carrying Neu5Acα2,6LacNAc residues by recombinant enzyme (Fig. 6). We tested the inhibitory effect of the artificial α2,6-sialoglycopolypeptide synthesized with recombinant enzyme on hemagglutination by SNA lectin, as a model of human influenza viruses. The α2,6-sialoglycopolypeptide prominently inhibited hemagglutination by SNA lectin with a very low concentration (IC_50 _0.94 nM) (Table 3). The α2,6-sialoglycopolypeptide displayed 780-fold higher affinities for the SNA hemagglutinins relative to the control fetuin (IC_50 _730 nM).

## Conclusion

The ST6Gal1 was purified, generating 2.2 mg purified fusion protein from only 11 silkworm larvae, with a recovery yield of 64%. The *N*-glycan patterns of purified ST6Gal1 were found to be approximately paucimannosidic type. The acceptor substrate specificity of the enzyme was similar to that of rat liver ST6Gal1. By catalytic reaction of this ST6Gal1, asialoglycopolypeptide was regioselectively and quantitatively sialylated to the terminal Gal residue to obtain α2,6-sialoglycopolypeptide. The α2,6-sialoglycopolypeptide selectively inhibited hemagglutination induced by SNA lectin, showing about 780-fold higher affinity than the control fetuin. This recombinant ST6Gal1 obtained using a silkworm expression system is a valuable tool for investigating the molecular mechanisms of biological and physiological events, such as cell-cell recognition and viral infections.

## Methods

### Construction of recombinant BmNPV bacmids

Rat ST6Gal1 (Δ1–189, lacking the *N*-terminal hydrophobic region) gene [29] was amplified from a rat liver cDNA library using the ST6Gal1 primer set (Table 4). Polymerase chain reaction (PCR) was performed according to the supplier's directions. All of the amplified PCR products were purified using a GFX PCR purification kit (Biocompare Inc., San Francisco, CA), digested with a *Bam*HI and *Eco*RI restriction kit and ligated using T4 ligase into pBlueBacHis2 (Invitrogen, Carlsbad, CA, USA) digested with *Bam*HI and *Eco*RI (pBH/ST6Gal1).

**Table 4:** Primers used for the cloning of rat ST6Gal1

Primers	Sequence (5'→3')
Forward primer	
CACC-bx	CACCATGAAGATACTCCTTGCTATTGCATTAATGTTG TCAACAGTAATGTGGGTGTCAACACAACCGCGGGGTT CTCATCATC
ST6Gal1	TATGGATCCGAGCAAGCAAGACCCTAAGGAAGACATT
CACC-bx-Strep-ST6Gal1	CACCATGAAGATACTCCTTGCTATTGCATTAATGTTG TCAACAGTAATGTGGGTGTCAACACCATGGAGCCATC CGCAGTTTGAAAAGAGCAAGCAAGACCCTAAGGAAG
CACC-bx-FLAG-ST6Gal1	CACCATGAAGATACTCCTTGCTATTGCATTAATGTTG TCAACAGTAATGTGGGTGTCAACACCAGACTACAAGG ATGACGATGACAAGAGCAAGCAAGACCCTAAGGAAG

Reverse primer	
ST6Gal1	TATGAATTCTCAACAACGAATGTTCCGGAAGCCAGA

In order to secrete expressed protein into the hemolymph of silkworm larvae, the signal sequence from bombyxin (bx) was added to ST6Gal1. Using pBH/ST6Gal1 as a template, PCR was carried out with CACC-bx forward and ST6Gal1 reverse primers (Table 4) with these conditions: 5 min at 94°C, 30 cycles at 94°C for 30 sec, 55°C for 40 sec, and 72°C for 1.5 min, followed by a final extension at 72°C for 5 min. The amplified DNA fragment was designated His-tagged ST6Gal1. Similarly, Strep-tagged and FLAG-tagged ST6Gal1 genes were amplified using the CACC-bx-Strep-ST6Gal1 and CACC-bx-FLAG-ST6Gal1 primer sets (Table 4). Using BmNPV bacmid-*CP*^-^-*Chi*^-^/bx-His-ST6Gal1 as a template, PCR was performed according to the supplier's directions. The DNA fragments were cloned into pENTR using pENTR Directional TOPO Cloning Kits (Invitrogen), resulting in pENTR/bx-His-, Strep- and FLAG-ST6Gal1. These were cloned into pDEST8 using the Gateway system (Invitrogen), resulting in pDEST8/bx-His-, Strep- and FLAG-ST6Gal1. To decrease proteolytic degradation of expressed ST6Gal1, a cysteine protease- and chitinase-deficient *B. mori *multiple nucleopolyhedrovirus (BmNPV-*CP*^-^-*Chi*^-^) bacmid [14] was used. The plasmid was transformed into *E. coli *DH10BacBm-*CP*^-^-*Chi*^-^, cultivated on LB plates containing kanamycin, gentamycin, tetracycline, chloramphenicol, 5-bromo-4-chloro-3-indolyl-β-D-galactopyranoside (X-gal) and isopropyl β-D-1-thiogalactopyranoside (IPTG). White colonies were picked, and positive clones were selected.

### Expression of ST6Gal1 in silkworm larvae

BmNPV bacmids DNA were injected directly into 1^st ^day fifth-instar hybrid Kinsyu × Syowa silkworm larvae (Ehime Sansyu, Co. Ltd., Yahatahama, Japan). BmNPV bacmid (100 ng/μl) and 10 1,2-dimyristyloxypropyl-3-dimethyl-hydroxy ethyl ammonium bromide (DMRIE)-C reagent (Invitrogen) were dissolved in 10 mM PBS (pH 7.4) and left to stand at room temperature for 45 min. Then, 40 μl of the mixture was injected into dorsal of the larvae using a syringe with a 26-gauge bevelled needle. The larvae were reared on an artificial diet (Silkmate 2S, Nihon Nosan Co. Ltd., Yokohama, Japan) at 27 ± 1°C. The expression of the fusion protein in silkworm larvae was confirmed using a sialyltransferase activity assay.

### Sample preparation for assay

At 1.5–7.5 days postinjection (d.p.i.) the larval hemolymph was harvested by cutting a caudal leg in a tube containing 1 mM 1-phenyl-2-thiourea, and centrifuged at 8000 rpm at 4°C for 10 min. The supernatant samples were immediately frozen at -80°C for future analysis.

### Sialyltransferase Assay

CMP-β-Neu5Ac (10 mM), 5-(5-dimethylaminonaphthalene-1-sulfonyl-2-(2-aminoethoxy))ethyl β-LacNAc (compound **3**, see Additional file 1) (5 mM), MnCl_2 _(2.5 mM) and BSA (2.5 mM) were dissolved in 50 mM MOPS (pH 7.4) followed by 13 μl of enzyme solution (total volume 20 μl) at 37°C. The reaction was initiated by addition of ST6Gal1. At each sample time, 2 μl of the reaction mixture was added to 98 μl of 25% acetonitrile. After filtration through a 0.45 μm nitrocellulose filter (Millipore, Bedford, MA) the filtrates were analyzed by HPLC using a TSK-gel ODS 80TsQA (4.6 × 250 mm, TOSOH Co.) column and eluted with 25% acetonitrile. A JASCO LC-2000 plus HPLC system equipped with an FP-2020 plus fluorescence detector (excitation, 330 nm; emission, 520 nm), operating isocratically at 1.0 ml/min at a column temperature of 40°C, was used. One unit of enzyme activity was defined as the amount of enzyme capable of catalyzing the transfer of 1 μmol of Neu5Ac per minute. The amount of protein was determined using a Bio-Rad protein assay kit.

### Purification of FLAG-tagged ST6Gal1 by affinity chromatography

All purification steps were performed at 4°C unless otherwise stated. At 6.5 d.p.i. the larval hemolymph was collected by cutting a caudal leg into a tube containing 1 mM 1-phenyl-2-thiourea, and centrifuging at 8000 rpm at 4°C for 10 min. The silkworm larval hemolymph (4.5 ml, 9.0 U) was twofold diluted with 50 mM MOPS buffer (pH 7.5) containing 150 mM NaCl and 0.02% Triton X-100. Proteins were precipitated in 25 ammonium sulphate. After centrifugation, solid ammonium sulphate was added to the supernatant to 70% saturation. The precipitate was dissolved with same buffer (6.9 ml, 7.2 U). After desalting with a Sephadex G-25M PD-10 column (Amersham Biosciences Corp., NJ, USA), the solution was applied to an anti-FLAG M2 (7 ml) column. The column was washed with 10 volumes of binding buffer 50 mM Tris-HCl buffer (pH 8.0) containing 150 mM NaCl and 0.02 Triton X-100, followed by 10 volumes of elute buffer 50 mM Tris-HCl buffer (pH 8.0) containing 150 mM NaCl, 0.02 Triton X-100 and 100 μg/ml FLAG peptide. Eluted fractions containing the purified enzyme were collected, dialyzed against 50 mM MOPS buffer (pH 7.5) containing 150 mM NaCl and 0.02 Triton X-100, and stored at 4°C (2.5 ml, 5.8 U) (Table 1).

### Characterization of recombinant ST6Gal1 expressed in silkworm larva

#### SDS-PAGE and Isoelectric Focusing

SDS-polyacrylamide gel electrophoresis (SDS-PAGE, 13%) was done by the method of Laemmli [33]. Samples were heated in the presence or absence of 2-mercaptoethanol at 100°C for 10 min. Gels were stained with Coomassie Brilliant Blue. The molecular masses on SDS-PAGE were estimated using recombinant ladder markers (10 to 100 kDa; XL-Ladder Low; APRO Life Science Institute, Inc., Japan). Isoelectric focusing (PhastGel IEF, pH 3–9) was done in a Phastsystem (GE Healthcare, UK). The pI standards (Pharmacia) used were trypsinogen (pI 9.30), lentil lectin basic band (8.65), lentil lectin acidic band (8.15), myoglobin basic band (7.35), myoglobin acidic band (6.85), human carbonic anhydrase B (6.55), bovine carbonic anhydrase B (5.85), β-lactoglobulin A (5.20), soybean trypsin inhibitor (4.55), and amyloglucosidase (3.50).

#### Molecular mass

The molecular mass of FLAG-tagged ST6Gal1 was determined by SDS-PAGE and MALDI-TOF mass spectroscopy. The MALDI-TOF mass spectrum was acquired on an AutoFlex (Bruker Daltonics, Germany) and measured in linear mode using 20-kV ion acceleration without postacceleration. The spectrum was recorded at a detector voltage of 1.65 kV and was the averaged results of at least 300 laser shots. SDHB was used as the matrix. Each sample was dissolved in 0.1% TFA: acetonitrile (2:1 v/v) and mixed with the matrix solution (1:4 v/v). The mixture (1 μl) was put on a stainless target and crystallized at room temperature. A mass calibration procedure was employed prior to the analysis of a sample using protein calibration standards I (Bruker Daltonics, Germany).

#### Effect of temperature and pH on enzyme activity and stability

The effect of temperature on SiaT activity was determined by incubating the purified enzyme with donor (CMP-Neu5Ac) and acceptor substrate (compound **3**) in 50 mM MOPS buffer (pH 7.4) containing MnCl_2 _and BSA for 15 min at different temperatures ranging from 4 to 80°C. Thermal stability of the enzyme was determined by assaying for residual enzyme activity after incubation at various temperatures for 30 min without substrates.

The optimal pH of SiaT activity was examined in the range of pH 2 to 10 under standard assay conditions using 50 mM of glycine-HCl buffer (pH 2 to 3), citrate buffer (pH 3 to 5), MES buffer (pH 5 to 6), MOPS buffer (pH 6 to 8), Tris-HCl buffer (pH 8 to 9) and glycine-NaOH buffer (pH 9 to 10). The effect of pH on ST6Gal1 stability was determined using the same buffer system in the range of pH 2 to 10. The enzyme solution was incubated at various pH values for 30 min at 4°C without substrates. The remaining enzyme activity was then measured at 37°C against donor (CMP-Neu5Ac) and acceptor substrate (compound **3**) in 50 mM MOPS buffer (pH 7.4) containing MnCl_2 _and BSA.

#### Kinetic parameters for the transfer reaction to acceptor substrates

CMP-β-Neu5Ac (10 mM), 5-(5-dimethylaminonaphthalene-1-sulfonyl-2-(2-aminoethoxy))ethyl β-LacNAc or -lactoside (compounds **3 **or **4**, see Additional file 1)(0.156 – 5.0 mM), MnCl_2 _(2.5 mM) and BSA (2.5 mM) were dissolved in 50 mM MOPS (pH 7.4) followed by addition of 80 μl of purified FLAG-tagged ST6Gal1 (total volume 100 μl) to initiate the reaction at 37°C. The reaction was initiated by addition of 2.5 μM of ST6Gal1. Samples (20 μl) were taken at intervals (0, 1, 2, 3, 4, 5 min) during the incubation, and inactivated with 80 μl of 25 acetonitrile. The amount of transfer product formed from initial substrates at an early stage was analyzed by HPLC. Quantification of products was as described above, and initial velocities (υ) were obtained directly from the initial slopes of the time-course plots. Six different substrate concentrations (0.156 – 5.0 mM) were used per experiment. The *V*_max _and *K*_m _values for synthetic acceptor substrates were calculated from the Lineweaver-Burk plot by the least squares method. Catalytic constant (*k*_cat_) of ST6Gal1 was defined as dividing *V*_max _by amount of ST6Gal1.

#### Characterization of N-linked glycan by HPLC mapping

The experimental procedures used, including the chromatographic and mass spectrometric conditions, have been described previously [34-36], with slight modifications in the preparation of 2-aminopyridine derivatives of the *N*-glycans. The purified FLAG-tagged ST6Gal1 (2.0 mg) was proteolyzed with trypsin and chymotrypsin, and further digested with PNGase F (New England Biolabs, MA, USA) to release *N*-glycans. After removal of the peptides by SepPack reversed-phase cartridges (Waters, MA, USA), the reducing ends of the *N*-glycans were derivatized with 2-aminopyridine (Wako, Osaka, Japan). The mixture of the 2-aminopyridine-derivatived (PA) glycans was separated by an octadecyl silica (ODS) column (Shimadzu, Kyoto, Japan) and the elution time recorded which represents the glucose unit (GU) value. The individual fractions were subjected to matrix-assisted laser desorption/ionization-time of flight mass spectrometry (MALDI-TOF MS). The identification of *N*-glycan structures was based on their GUs and mass values in comparison with PA-glycans in the web application GALAXY database http://www.glycoanalysis.info/galaxy2/ENG/systemin1.jsp[37]. The PA-oligosaccharides were subject to matrix-assisted laser desorption/ionization time of flight mass spectrometry (MALDI-TOF-MS) analyses and co-chromatographed with reference to PA-oligosaccharides on the columns to confirm their identities.

### Synthesis of sialoglycopolypeptides as glycoprotein mimetics using recombinant ST6Gal1

α2,6-Sialoglycopolypeptide was enzymatically synthesized from asialoglycopolypeptide [19] using recombinant ST6Gal1. A mixture containing 5.0 mg of asialoglycopolypeptide, 16.0 mM CMP-β-Neu5Ac, 80 mU/ml of the purified FLAG-tagged ST6Gal1, 2.5 mM MnCl_2_, 0.1 BSA and 10 U/ml of calf intestine alkaline phosphatase (Boehringer-Mannheim, Mannheim, Germany) in 50 mM MOPS buffer (pH 7.4) was incubated at 37°C for 48 h in a total volume of 1.0 ml. After heating at 100°C followed by centrifugation, the supernatant from the reaction mixture was directly loaded onto a Sephadex G-25M PD-10 column equilibrated with 100 mM PBS (pH 7.4). The high-molecular-weight fraction was dialyzed against distilled water for 3 days and lyophilized to yield α2,6-sialoglycopolypeptide (6.0 mg) (Fig. 2).

### Hemagglutination inhibition assay

The hemagglutination inhibition assay was carried out using 96-well microtiter plates as described previously [38]. Phosphate-buffered saline (PBS, pH 6.5) was used as a dilution buffer. SNA (EY Laboratories Inc., San Mteo, CA, USA) lectin suspension (2^2 ^hemagglutination titres in 0.025 ml of PBS) was added to each well containing the artificial glycopolypeptides (200 to 0.024 μnM) or fetuin (250 to 0.122 M) in a twofold serial dilution in dilution buffer. After incubation for 1 h at 4°C, 0.05 ml of 0.6 (v/v) guinea-pig suspension erythrocytes was added to the plates, and allowed to settle for 2 h at 4°C. The maximum dilution of the samples showing complete inhibition of hemagglutination was defined as the hemagglutination inhibition titer.

## Abbreviations

ABEE: ethyl 4-aminobenzoate; BmNPV: *Bombyx mori *nucleopolyhedrovirus; bx: bombyxin; CMP: cytidine monophosphate; DMB: 1,2-diamino-4,5-methylenedioxybenzene; d.p.i.: days post-injection; Gal: galactose; GlcNAc: *N*-acetylglucosamine; IPTG: isopropyl-1-thio-β-D-galactopyranoside; Lac: lactose; LacNAc: *N*-acetyllactosamine; Neu5Ac: *N*-acetylneuraminic acid; PCR: polymerase chain reaction; ST6Gal1: α2,6-sialyltansferase; SNA: *Sambucus nigra*; X-gal: 5-bromo-4-chloro-3-indolyl-β-D-galactopyranoside.

## Authors' contributions

MO carried out the experimental design, purification of ST6Gal1, the synthesis of sialoglycopolypeptide, and bioassay. MN participated in genetic manipulation, expressing ST6Gal1, rearing silkworm and sample collection from silkworm. TK participated in the experimental design and genetic manipulation. TO synthesized the substrate for ST6Gal1 activity assay. HY and KK participated in characterization of *N*-linked glycan. TU participated in the experimental design of Sialoglycopolypeptide synthesis and discussion. EYP directly supervised the project, participated in its experimental design, data interpretation, and was responsible for writing the manuscript. All authors have read and approved the manuscript.

## Supplementary Material

Additional file 1 Chemo-enzymatic synthesis of fluorescent-labelled acceptor substrates and Neu5Acα2,6LacNAc glycoside. Click here for file
