# Supplementary material for: Synthesis of sialoglycopolypeptide for potentially blocking influenza virus infection using a rat α2,6-sialyltransferase expressed in BmNPV bacmid-injected silkworm larvae
Source: BMC Biotechnol. 2009 Jun 5;9:54. doi: 10.1186/1472-6750-9-54 (PMC3224744; doi:10.1186/1472-6750-9-54)
Supplement: Additional file 1 — Chemo-enzymatic synthesis of fluorescent-labelled acceptor substrates and Neu5Acα2,6LacNAc glycoside. [file 1472-6750-9-54-S1.doc]

# **Chemo-enzymatic Synthesis of Fluorescent-labeled Acceptor Substrates and Neu5Acα2,6LacNAc Glycoside**

***1. Chemo-enzymatic Synthesis of Fluorescent-labeled Acceptor Substrates on Sialyltransferase (Compounds 3 and 4)***

The crude cellulase from *Trichoderma reesei* was partially purified by removing unwanted β-D-galactosidase by our previously described method (Ogata et al., 2007; Yasutake et al., 2003). Partially purified enzyme (4000 U of Lac β-*p*NP hydrolytic activity) in 49 ml of 100 mM sodium acetate buffer (pH 4.0) was added to a mixture containing 65 mmol of LacNAc, 81 mmol of 2-(2-trifluoroacetamidoethoxy)ethanol. After, reaction mixture was incubated at 40°C for 144 h, it was terminated by heating at 100°C for 10 min. The supernatant obtained from centrifugation (17,000 rpm, 10 min) was loaded onto a charcoal-Celite column (4.5 × 100 cm) equilibrated with distilled water. Subsequently, the adsorbed portion was eluted with a linear gradient of 0% (3 L) to 15% ethanol (3 L), followed by 50% ethanol, at a flow rate of 5.0 ml/min, and a fraction size of 60 ml/tube. The neutral sugar content of the eluted fractions was measured at 485 nm by the phenol-sulfuric acid method. An aliquot from fractions 33-89 was concentrated and lyophilized: LacNAc was recovered in a yield of 92% (23 g). An aliquot from fractions 90-135 was then concentrated and dissolved in 15 ml of CHCl_3_/CH_3_OH/H_2_O=7/3/0.5 and then loaded onto a Silica Gel 60N column (4.0 × 50 cm). The column was developed with the same solvent at a flow rate of 10 ml/min and a fraction size of 25 ml/tube. An aliquot from fractions 75-95 was then concentrated and lyophilized: 2-(2-trifluoroacetamidoethoxy)ethyl β-LacNAc **1** was obtained in a total yield of 1.0% (380 mg) based on LacNAc added (Supplementary Fig. 1). The HRESIMS spectra were measured on a JMS-T100LC mass spectrometer. ^1^H and ^13^C NMR spectra were recorded on a JEOL JNM-LA 500 spectrometer (JEOL Co. Ltd., Akishima, Japan) at 25°C. Chemical shifts are expressed in δ relative to sodium 3-(trimethylsilyl) propionate as an external standard.

HRESIMS: *m/z* 589.18333 [M + Na]^+^ (calcd for C_20_H_33_F_3_N_2_Na_1_O_13_, 589.18324); ^1^H-NMR (D_2_O, 500 MHz): δ 4.59 (d, 1H, *J*_1, 2_ 8.0 Hz, H-1), 4.49 (d, 1H, *J*_1', 2'_ 7.7 Hz, H-1'), 4.03-3.97 (2H, H-6b, H-αb), 3.94 (1H, H-4'), 3.84 (dd, 1H, *J*_5, 6a_ 5.2, *J*_6a, 6b_ 12.2 Hz, H-6a), 3.80-3.66 (12H, H-6', H-5', H-3', H-4, H-3, H-2, H-αa, H-β, H-γ), 3.60 (1H, H-5), 3.58-3.52 (3H, H-2', H-ω), 2.05 (s, 3H, CH_3_CONH-); ^13^C-NMR (D_2_O, 500 MHz): δ 177.3 (CH_3_CONH-), 161.9 (CF_3_CONH-), 118.7 (CF_3_CONH-), 105.7 (C-1'), 103.8 (C-1), 81.3 (C-4), 78.2 (C-5'), 77.6 (C-5), 75.4 (C-3), 75.3 (C-3'), 73.8 (C-2'), 72.4 (C-β), 71.8 (C-α), 71.4 (C-4'), 71.0 (C-γ), 63.8 (C-6'), 62.9 (C-6), 57.9 (C-2), 42.2 (C-ω), 25.0 (CH_3_CONH-). 2-(2-Trifluoroacetamidoethoxy)ethyl β-lactoside **2** was synthesized in a similar manner from lactose and 2-(2-trifluoroacetamidoethoxy)ethanol (Supplementary Fig. 1).

Next, Compound **1** (100 mg, 0.18 mmol) was dissolved in 1.0 M NaOH (1 ml). After the mixture was incubated for 60 min at room temperature, it was loaded onto a Sephadex G-25 column (2.5 × 55 cm) equilibrated with water at a flow rate of 0.4 ml/min and fraction size of 3.0 ml/tube. An aliquot from fractions 42-47 was concentrated and lyophilized. 2-(2-aminoethoxy)ethyl β-LacNAc was obtained at a total yield of 96% (80 mg). The resulting compound (50 mg, 0.11 mmol) and Na_2_CO_3_ (22.5 mg, 0.2 mmol) were dissolved in 65% acetone (19.2 ml). Dansyl chloride (29 mg, 0.11 mmol) was added to the solution with continuous stirring at room temperature for 4 h. The reaction was neutralized with 1M HCl and concentrated to a syrup before dissolving in 1.0 ml of 30% acetonitrile and loading onto an ODS column (2 × 50 cm). The column was developed with the same solvent at a flow rate of 2.5 ml/min and a fraction size of 27 ml/tube. Fractions 9-12 were pooled and concentrated: 5-(5-dimethylaminonaphthalene-1-sulfonyl-2-(2-aminoethoxy))ethyl β-LacNAc **3** was obtained in a total yield of 71% (53 mg) (Supplementary Fig. 1). HRESIMS: *m/z* 726.25204 [M + Na]^+^ (calcd for C_30_H_45_N_3_Na_1_O_14_S_1_, 726.25199); ^1^H-NMR (D_2_O, 500 MHz): δ 8.28 (1H, H-d), 8.22 (1H, H-i), 8.11 (1H, H-b), 7.51 (1H, H-h), 7.44 (1H, H-c), 7.12 (1H, H-g), 4.47 (d, 1H, *J*_1, 2_ 7.6 Hz, H-1), 4.39 (d, 1H, *J*_1', 2'_ 6.8 Hz, H-1'), 3.96-3.92 (2H, H-6b, H-4'), 3.82 (dd, 1H, *J*_5, 6a_ 4.9, *J*_6a, 6b_ 12.2 Hz, H-6a), 3.80-3.66 (7H, H-6', H-5', H-3', H-4, H-3, H-2), 3.59-3.54 (2H, H-2', H-αb), 3.50 (1H, H-5), 3.30 (1H, H-αa), 3.19 (2H, H-γ), 3.07 (2H, H-β), 2.99 (2H, H-ω), 2.61 (s, 6H, (CH_3_)_2_N-), 1.95 (s, 3H, CH_3_CONH-); ^13^C-NMR (D_2_O, 500 MHz): δ 177.0 (CH_3_CONH-), 153.7 (C-f), 137.3 (C-a), 132.8 (C-d), 132.0 (C-b), 131.8 (C-e, C-j), 131.4 (C-h), 126.6 (C-c), 121.8 (C-i), 118.5 (C-g), 105.7 (C-1'), 103.7 (C-1), 81.3 (C-4), 78.2 (C-5'), 77.5 (C-5), 75.4 (C-3), 75.2 (C-3'), 73.8 (C-2'), 72.0 (C-β), 71.43 (C-α), 71.38 (C-4'), 71.5 (C-γ), 63.8 (C-6'), 62.9 (C-6), 57.8 (C-2), 47.7 ((CH_3_)_2_N-), 45.0 (C-ω), 25.1 (CH_3_CONH-). 5-(5-Dimetylaminonaphthalene-1-sulfonyl-2-(2-aminoethoxy))ethyl β-lactoside **4** was synthesized from **2** in a similar manner (Supplementary Fig. 1).

***2. Synthesis of Fluorescent-labeled Neu5Acα2,6LacNAc Glycoside as a Transfer Product using Recombinant ST6Gal1 (Compound 5)***

5-(5-Dimetylaminonaphthalene-1-sulfonyl-2-(2-aminoethoxy)) ethyl β-Neu5Acα2,6LacNAc (compound **5**) was synthesized by the alternative addition of α2,6 linked-Neu5Ac to compound **3** using recombinant ST6Gal1 (Supplementary Fig. 1). Sixty mU/ml of the crude FLAG-tagged ST6Gal1, 2.5 mM MnCl_2_, 0.1% BSA and 10 U/ml of calf intestine alkaline phosphatase (Boehringer-Mannheim, Mannheim, Germany) in 50 mM MOPS buffer (pH 7.4) was added to a mixture containing 30 mg of compound **3**, 16 mM CMP-β-Neu5Ac. The reaction mixture was incubated at 37°C for 24 h in a total volume of 5.4 ml. After reaction was terminated by boiling for 5 min, the resulting precipitate was removed by centrifugation (8000 g, 5 min) and the supernatant was loaded onto an ODS column (2.0 × 30 cm) equilibrated with 10% CH_3_CN. The column was developed with the same solvent at a flow rate of 1.5 ml/min and a fraction size of 10 ml/tube. The eluate was monitored by measuring the absorbance at 210 nm and 300 nm using a spectrophotometer. An aliquot from fractions 21-47 was then concentrated and lyophilized: compound **5** was obtained in a total yield of 87% (37 mg) based on the acceptor substrate **3** added (Supplementary Fig. 1). HRESIMS: *m/z* 993.35411 [M − H]^−^ (calcd for C_41_H_61_N_4_O_22_S_1_, 993.34981); ^1^H-NMR (D_2_O, 500 MHz): δ 8.42 (1H, H-d), 8.25 (1H, H-i), 8.20 (1H, H-b), 7.65 (1H, H-h), 7.61 (1H, H-c), 7.33 (1H, H-g), 4.44 (d, 1H, *J*_1', 2'_ 8.0 Hz, H-1'), 4.42 (d, 1H, *J*_1, 2_ 8.0 Hz, H-1), 4.00 (1H, H-6'b), 3.95 (1H H-6b), 3.93 (1H, H-4'), 3.91-3.86 (2H, H-8'', H-9''b), 3.83-3.78 (3H, H-5'', H-5', H-6a), 3.74-3.52 (12H, H-9''a, H-7'', H-6'', H-4'', H-6'a, H-3', H-2', H-5, H-4, H-3, H-2, H-αb), 3.30-3.22 (3H, H-αa, H-γ), 3.08-3.06 (4H, H-β, H-ω), 2.80 (s, 6H, (CH_3_)_2_N-), 2.69 (dd, 1H, *J*_3''ax, 3''eq_ 12.5, *J*_3''eq, 4''_ 4.5 Hz, H-3''eq), 2.04 (s, 3H, CH_3_CONH-''), 1.98 (s, 3H, CH_3_CONH-), 1.71 (t, 1H, *J*_3''ax, 3''eq_ 12.5, *J*_3''ax, 4''_ 12.5 Hz, H-3''ax); ^13^C-NMR (D_2_O, 500 MHz): δ 177.8 (CH_3_CONH-''), 177.2 (CH_3_CONH-), 176.3 (HOOC-''), 153.6 (C-f), 137.1 (C-a), 132.8 (C-d), 132.3 (C-b), 131.7 (C-e, C-j), 131.6 (C-h), 126.8 (C-c), 121.9 (C-i), 118.8 (C-g), 106.3 (C-1'), 103.6 (C-1), 103.0 (C-2''), 83.5 (C-4), 77.3 (C-5), 76.5 (C-5'), 75.4 (C-6''), 75.31 (C-3), 75.26 (C-3'), 74.6 (C-8''), 73.6 (C-2'), 72.0 (C-β), 71.5 (C-γ), 71.4 (C-α), 71.3 (C-4'', C-4'), 71.0 (C-7''), 66.2 (C-6'), 65.5 (C-9''), 63.2 (C-6), 57.6 (C-2), 54.8 (C-5''), 47.8 ((CH_3_)_2_N-), 45.0 (C-ω), 43.0 (C-3''), 25.1 (CH_3_CONH-), 24.9 (CH_3_CONH-'').

**Results**

**Chemo-enzymatic Synthesis of Fluorescent-labeled Acceptor Substrates on Sialyltransferase**

Compounds **1** and **2** were enzymatically synthesized by a condensation reaction between LacNAc/lactose and 2-(2-trifluoroacetamidoethoxy)ethanol using cellulase from *T. reesei* (Supplementary Fig. 1). Compounds **1** and **2** with aglycon trifluoroacetamido group were then deacylated to 2-(2-aminoethoxy)ethyl β-LacNAc or -lactoside by hydrolysis in an alkaline solution, respectively. The resulting amino function was coupled to dansyl chloride in acetone to produce fluorescently-labeled-LacNAc/ -lactose derivatives (**3** and **4)** (Supplementary Fig. 1). Compounds **3** and **4** were easily purified with an ODS column, in yields of 71% and 68% based on the corresponding glycosides, respectively.

**Supplementary Figure 1.** (1) Chemo-enzymatic synthesis of fluorescent-labeled acceptor substrates on sialyltransferase and (2) sialyltransferase activity assay method
